# Multiplex Real-Time PCR for Detection of *Staphylococcus aureus*, *mecA* and Panton-Valentine Leukocidin (PVL) Genes from Selective Enrichments from Animals and Retail Meat

**DOI:** 10.1371/journal.pone.0097617

**Published:** 2014-05-21

**Authors:** Valeria Velasco, Julie S. Sherwood, Pedro P. Rojas-García, Catherine M. Logue

**Affiliations:** 1 Department of Veterinary and Microbiological Sciences, North Dakota State University, Fargo, North Dakota, United States of America; 2 Department of Animal Sciences, University of Concepción, Chillán, Chile; 3 Laboratory of Animal Physiology and Endocrinology, Veterinary Sciences, University of Concepción, Chillán, Chile; 4 Department of Veterinary Microbiology and Preventive Medicine, College of Veterinary Medicine, Iowa State University, Ames, Iowa, United States of America; Columbia University, College of Physicians and Surgeons, United States of America

## Abstract

The aim of this study was to compare a real-time PCR assay, with a conventional culture/PCR method, to detect *S. aureus*, *mecA* and Panton-Valentine Leukocidin (PVL) genes in animals and retail meat, using a two-step selective enrichment protocol. A total of 234 samples were examined (77 animal nasal swabs, 112 retail raw meat, and 45 deli meat). The multiplex real-time PCR targeted the genes: *nuc* (identification of *S. aureus*), *mecA* (associated with methicillin resistance) and PVL (virulence factor), and the primary and secondary enrichment samples were assessed. The conventional culture/PCR method included the two-step selective enrichment, selective plating, biochemical testing, and multiplex PCR for confirmation. The conventional culture/PCR method recovered 95/234 positive *S. aureus* samples. Application of real-time PCR on samples following primary and secondary enrichment detected *S. aureus* in 111/234 and 120/234 samples respectively. For detection of *S. aureus*, the *kappa* statistic was 0.68–0.88 (from substantial to almost perfect agreement) and 0.29–0.77 (from fair to substantial agreement) for primary and secondary enrichments, using real-time PCR. For detection of *mecA* gene, the *kappa* statistic was 0–0.49 (from no agreement beyond that expected by chance to moderate agreement) for primary and secondary enrichment samples. Two pork samples were *mecA* gene positive by all methods. The real-time PCR assay detected the *mecA* gene in samples that were negative for *S. aureus*, but positive for *Staphylococcus* spp. The PVL gene was not detected in any sample by the conventional culture/PCR method or the real-time PCR assay. Among *S. aureus* isolated by conventional culture/PCR method, the sequence type ST398, and multi-drug resistant strains were found in animals and raw meat samples. The real-time PCR assay may be recommended as a rapid method for detection of *S. aureus* and the *mecA* gene, with further confirmation of methicillin-resistant *S. aureus* (MRSA) using the standard culture method.

## Introduction

*Staphylococcus aureus* is considered as an important cause of a wide variety of diseases in humans such as: food poisoning, pneumonia, wound and nosocomial infections [1], [2]. There are many anti-staphylococcal agents; however, the bacterium has developed mechanisms to neutralize them such as the methicillin resistance mechanism [3]. Methicillin-resistant *S. aureus* (MRSA) is an increasing cause of health care-associated (HA-MRSA) [1], community-associated (CA-MRSA) [2], and livestock-associated (LA-MRSA) infections worldwide [4].

The altered penicillin-binding protein (PBP2a) is associated with methicillin resistance. This protein has a reduced affinity for β-lactam antibiotics [5], [6], and is encoded by the *mecA* gene, which is carried on the staphylococcal cassette chromosome *mec* (SCC*mec*) [5]. CA-MRSA strains are more likely to encode the Panton–Valentine leukocidin (PVL) toxin, which is a pore-forming toxin considered as a virulence factor [7], [8]. The PVL toxin has been related to life-threatening CA-MRSA infections and deaths, primarily severe skin infections and tissue necrosis [9].

In the United States, approximately 29% (78.9 million people) and 1.5% (4.1 million) of the population were estimated to be nasal carriers of *S. aureus* and MRSA, respectively [10]. An estimated 478,000 hospitalizations corresponded to *S. aureus* infections, of which 278,000 hospitalizations were attributed to MRSA infections in 2005 [11]. In addition, the carriage of MRSA in meat-producing animals [12]–[14] and the contamination of retail meat with MRSA [15]–[17] have increased the concern that food may serve as a vehicle to transmit MRSA to the human population [17].

Different culture methods have been used to detect MRSA. Generally, conventional microbiological procedures are laborious, since they require the isolation of *S. aureus* before assessing methicillin resistance. However, culture methods are still considered as standard methods for traditional confirmation of *S. aureus*. Wertheim *et al*. (2001) [18] developed a selective media containing phenol red and antibiotics (aztreonam and ceftizoxime), increasing the sensitivity of the detection of MRSA after 48 h of incubation, but at the expense of longer time needed for confirmation. The isolation and identification of MRSA, including selective enrichment and plating on selective agars, followed by confirmation using biochemical testing and/or PCR assays, requires 3–7 days approximately [15], [16], [19]. Therefore, development of a rapid method for detection of MRSA has become an important need in the microbiological analysis of samples especially those where there is a potential risk of exposure for humans.

Real-time PCR technology has been used as an alternative to culture methods for the rapid detection of *S. aureus* and MRSA. Detection using real-time PCR may decrease the time of analysis to 18 h after consecutive broth enrichment in clinical samples [20]; or <2 h in positive blood cultures [21], [22]. However, most studies have used real-time PCR to detect MRSA in clinical samples and isolates and a few studies have evaluated the application of this method for the detection of MRSA in animals [23], [24] and meat [15], [25], [26].

Since *S. aureus* and MRSA have been found in food-producing animals and retail meat, increasing the concern about the exposure for humans through the food chain, and there is a need to decrease the time of analysis, we analyzed samples obtained from animals and retail meats using primary and secondary selective enrichments in order to detect *nuc* (identification of *S. aureus*), *mecA* (associated with methicillin resistance) and PVL (virulence factor) genes using a multiplex real-time PCR assay. The results obtained with the real-time PCR assay were compared with the results from a culture method, considered as the standard method, which also included the two-step selective enrichment, followed by selective plating, biochemical testing and conventional multiplex PCR. Positive samples obtained with the culture method were characterized by multilocus sequence typing (MLST) and the antimicrobial resistance profiles were obtained.

## Materials and Methods

### Samples

A total of 77 nasal swabs (Becton, Dickinson and Company, Sparks, MD, USA) were collected from animals (sheep, n = 35; pigs, n = 28; cows, n = 14) sampled immediately after stunning at the Meat Lab (Department of Animal Sciences); and at the ND Veterinary Diagnostic Lab at North Dakota State University, Fargo, ND. Animal samples were collected during the period May 2010-April 2011. The protocol of sampling was approved by the North Dakota State University Institutional Biosafety Committee (B10014).

In addition, 112 retail raw meat (pork, n = 39; chicken, n = 37; beef, n = 36) and 45 deli meat (ham, n = 20; turkey, n = 16; chicken, n = 9) samples were randomly purchased from four different supermarket chains in Fargo, ND. Sampling visits were made between June 2010 and January 2011. All samples were immediately stored at 4°C and processed within six hours of collection.

### Culture method

*Staphylococcus aureus* were isolated by the two-step selective enrichment procedure according to the method described by de Boer *et al*. (2009) [15] followed by plating steps on selective agar. Briefly, for the primary enrichment, a 25 g sample of retail meat and 225 mL of MHB+6.5%NaCl (Mueller-Hinton broth [Difco, Becton, Dickinson, Sparks, MD, USA] with added 6.5% sodium chloride [VWR International, West Chester, PA, USA]) were placed in a sterile stomacher bag and homogenized using a stomacher400 circulator (Seaward, England) at 230 rpm for 90 seconds. The suspension was incubated for 18–20 h at 37°C. Following primary enrichment, a secondary enrichment was used by inoculating 1 mL of the primary enrichment broth into 9 mL of PHMB^+^ (D-mannitol in phenol red mannitol broth base [Difco, Becton, Dickinson, Sparks, MD, USA] containing ceftizoxime [5 µg mL^−1^, US Pharmacopeia, Rockville, MD, USA] and aztreonam [75 µg mL^−1^, Sigma Chemical CO., Louis, MO, USA] according to Wertheim *et al*. [2001] [18]), followed by incubation for 18–20 h at 37°C. Nasal swabs from animals were placed directly in 9 mL MHB+6.5%NaCl and incubated for 18–20 h at 37°C. Then, the secondary enrichment was used following the procedure described above.

Following incubation of the secondary enrichment broth, all samples were struck directly to BP medium (Baird-Parker medium [Difco, Becton, Dickinson, Sparks, MD, USA]) supplemented with egg yolk tellurite according to manufacturer's recommendations and incubated for 48 h at 37°C. Presumptive *S. aureus* colonies (black colonies surrounded by 2 to 5 mm clear zones) were transferred to TSA II 5%SB plates (Trypticase soy agar with 5% sheep blood [Difco, Becton, Dickinson, Sparks, MD, USA]) and incubated for 18–20 h at 37°C. Suspect *S. aureus* colonies (presence of β-haemolysis) were confirmed using Sensititre Gram Positive ID (GPID) plates (Sensititre, TREK Diagnostic Systems Ltd., Cleveland, OH, USA) according to the manufacturer's instructions.

### Conventional multiplex PCR method

Confirmed *S. aureus* strains were recovered from frozen stock to TSA plates (Trypticase soy agar [Difco, Becton, Dickinson, Sparks, MD, USA]) and incubated at 37°C for 18–24 h. DNA extraction was carried out by suspending one colony in 50 µL of DNase/RNase-free distilled water (Gibco Invitrogen, Grand Island, NY, USA), heating (99°C, 10 min) and centrifugation (30,000×*g*, 1 min) to remove cellular debris. The remaining DNA was transferred to a new tube and stored at −20°C.

A multiplex PCR assay for the detection of 16S rRNA (identification of *S. aureus*), *mecA* (associated with methicillin resistance) and PVL-encoding genes (virulence factor) (Table 1) included 2 µL of the DNA template (described above) added to a 50 µL final reaction mixture containing: 1X Go Taq Reaction Buffer (pH 8.5), 0.025 µL^−1^ of Go Taq DNA polymerase, 200 µM dNTP (Promega, Madison, WI, USA) and 1 µM of primers (16S rRNA, *mecA*, LukS/F-PV) (Integrated DNA Technologies, Inc., Coralville, IA, USA).

**Table 1:** Nucleotide sequence of the primers and probes used in conventional multiplex PCR, multiplex real-time PCR.

Primer or probe name	Sequence (5′→3′)	5′ Reporter dye 3′ Quencher
16S rRNA*		
Staph-756F	AAC TCT GTT ATT AGG GAA GAA CA	
Staph-750R	CCA CCT TCC TCC GGT TTG TCA CC	
*nuc* †		
*nuc* For	CAA AGC ATC AAA AAG GTG TAG AGA	
*nuc* Rev	TTC AAT TTT CTT TGC ATT TTC TAC CA	Texas Red
*nuc* Probe	TTT TCG TAA ATG CAC TTG CTT CAG GAC CA	Iowa Black
*mecA*		
*mecA*-1F*	GTA GAA ATG ACT GAA CGT CCG ATA A	
*mecA*-2F*	CCA ATT CCA CAT TGT TTC GGT CTA A	
*mecA* For†	GGC AAT ATT ACC GCA CCT CA	
*mecA* Rev†	GTC TGC CAC TTT CTC CTT GT	FAM†
*mecA* Probe†	AGA TCT TAT GCA AAC TTA ATT GGC AAA TCC	TAMRA†
PVL		
*luk*-PV-1F*	ATC ATT AGG TAA AAT GTC TGG ACA TGA TCC A	
*luk*-PV-2R*	GCA TCA AGT GTA TTG GAT AGC AAA AGC	
PVL For†	ACA CAC TAT GGC AAT AGT TAT TT	
PVL Rev†	AAA GCA ATG CAA TTG ATG TA	Cy5†
PVL Probe†	ATT TGT AAA CAG AAA TTA CAC AGT TAA ATA TGA	Iowa Black

*Conventional multiplex PCR, according to McClure *et al*. (2006) [43].

† Multiplex real-time PCR, according to McDonald *et al*. (2005) [35].

Multiplex PCR reactions were carried out in a thermocycler (Eppendorf, Hamburg, Germany), and the PCR conditions were adjusted according to the protocol described by Makgotlho *et al*. (2009) [27] as follows: initial denaturation at 94°C for 10 min, followed by 10 cycles of denaturation at 94°C for 45 s, annealing at 55°C for 45 s and extension at 72°C for 75 s followed by another 25 cycles of 94°C for 45 s, 50°C for 45 s and a final extension step at 72°C for 10 min. An external positive control (DNA extracted from MRSA ATCC 35591, positive for *mecA* and PVL genes) and an external negative control (DNase/RNase-free distilled water) were included with each run.

PCR amplicons (10 µL) were loaded into a 1.5% (wt/vol) agarose gel (Agarose I™) using EzVision One loading dye (Amresco, Solon, OH, USA) and electrophoresis was carried out in 1X TAE buffer at 100 v for 1 h. A molecular weight marker 100-bp ladder (Promega, Madison, WI, USA) were included on each gel. Bands were visualized using an Alpha Innotech UV imager (FluorChem™).

### Multiplex real-time PCR assay

DNA was extracted from the primary and secondary enrichment broths of the animal and meat samples using the boiling method described previously by de Medici *et al*. (2003) [28]. Five microliters of DNA template extracted was used in the real-time iQ™ Multiplex Powermix (Bio-Rad Laboratories, Hercules, CA, USA), in a final volume of 20 µL per reaction.

The real-time PCR assay targeted the following genes: *nuc* (identification of *S. aureus*), *mecA* (associated with methicillin resistance) and PVL-encoding genes (virulence factor) (Table 1).

The final concentrations in the reaction mixture were: 300 nM of primers (forward and reverse), 200 nM of fluorogenic probes (Applied Biosystems, Foster City, CA, USA), and 1X iQ™ Multiplex Powermix (Bio-Rad Laboratories, Hercules, CA, USA), according to the manufacturer's recommendations.

The thermal cycling conditions were adjusted to an initial denaturation of 3 min at 95°C, followed by 40 PCR cycles of 95°C for 15 s and 55°C for 1 min, using an iCycler IQ™ real time PCR system (Bio-Rad Laboratories, Hercules, CA, USA). An external positive control (DNA extracted from MRSA ATCC 35591, positive for mecA and PVL genes) and an external negative control (DNase/RNase-free distilled water) were included with each plate. Data analysis was carried out using the iCycler software version 3.0 (Bio-Rad Laboratories, Hercules, CA, USA).

### Characterization of *S. aureus* strains isolated by culture method

#### Multilocus Sequence typing (MLST)

Briefly, *S. aureus* isolates were struck to TSA plates and incubated at 37°C for 18–24 h. Colonies were picked to 40 µL of single cell lysing buffer (50 µg/mL of Proteinase K, Amresco; in TE buffer [pH = 8]), and then lysed by heating to 80°C for 10 min followed by 55°C for 10 min in a thermocycler. The final suspension was diluted 1∶2 in sterile water, centrifuged to remove cellular debris, and transferred to a sterile tube (Marmur, 1961) [29]. The housekeeping genes: *arcC*, *aroE*, *glpF*, *gmk*, *pta*, *tpi*, and *yqiL*, were amplified [30]. All PCR reactions were carried out in 50-µL volumes: 1 µL of DNA template, Taq DNA polymerase (Promega) (1.25 U), 1X PCR buffer (Promega), primers (0.1 µM) (Integrated DNA Technologies, Inc.), and dNTPs (200 µM) (Promega). PCR settings were adjusted according to Enright et al. (2000) [30] using a thermocycler (Eppendorf). Ten microliters of the PCR products were loaded into 1% agarose gels in 1X TAE with EzVision One loading dye, and run at 100V in 1X TAE for 1 h. Images were captured using an Alpha Innotech imager. After PCR, each amplicon was purified of amplification primer using the QIAquickPCR Purification Kit (Qiagen, Valencia, CA) as per manufacturer's instructions. Purified DNA was sequenced at Iowa State University's DNA Facility (Ames, IA) using an Applied Biosystems 3730xl DNA Analyzer (Applied Biosystems, Foster City, CA). Sequence data were imported into DNAStar (Lasergene, Madison, WI), trimmed, and aligned to the control sequences (from the MLST site) and interrogated against the MLST database (http://saureus.mlst.net/). Sequence types were added to the strain information for analysis in BioNumerics.

#### Resistance profiles

The antimicrobial resistance profiles (AR) of *S. aureus* isolates (n = 95) were determined using the broth microdilution method (CMV3AGPF, Sensititre, Trek Diagnostics), according to the manufacturer's and the National Antimicrobial Resistance Monitoring System (NARMS) guidelines for animal isolates [31]. Antimicrobials in the panel and their resistance breakpoints were as follows: erythromycin (≥8 µg/mL), tetracycline (≥16 µg/mL), ciprofloxacin (≥4 µg/mL), chloramphenicol (≥32 µg/mL), penicillin (≥16 µg/mL), daptomycin (no interpretative criteria), vancomycin (≥32 µg/mL), nitrofurantoin (≥128 µg/mL), gentamicin (>500 µg/mL), quinupristin/dalfopristin (≥4 µg/mL), linezolid (≥8 µg/mL), kanamycin (≥1024 µg/mL), tylosin (≥32 µg/mL), tigecycline (no interpretative criteria), streptomycin (>1000 µg/mL), and lincomycin (≥8 µg/mL). Resistance to at least three classes of antibiotics was considered as multidrug resistance (MDR) [32].

### Statistical analysis

The 95% confidence intervals for prevalence were obtained, using the plus four estimate when positive or negative samples were less than 15. The Chi-square test was used to assess the significance in proportion of positive samples between sample types, only if no more than 20% of the expected counts were less than 5 and all individual expected counts were 1 or greater [33]. On the contrary, Fisher's exact test was used with two-sided p-values. SAS software version 9.2 (SAS Institute Inc., Cary, NC) was used to assess significance with a *P*<0.05.

As there is no true gold standard method for *S. aureus* and MRSA detection, the *kappa* statistic was calculated to compare agreement between real-time PCR assay (using primary and secondary enrichment) and conventional culture/PCR method.

## Results

The culture method included a biochemical identification to confirm *S. aureus*, which agreed with the results of the conventional multiplex PCR that detected the gene 16S rRNA. This method detected 95 positive *S. aureus* samples from a total of 234 samples collected (Table 2). The multiplex real-time PCR assay using primary and a secondary enrichment samples, recovered *S. aureus* (detection of *nuc* gene) from 111 and 120 samples of 234 samples respectively.

**Table 2:** Detection of *S. aureus*, *mecA* and PVL genes from animals and retail meat using a conventional culture/PCR method and a real-time PCR assay.

Sample type	No. of samples				Real-time PCR					
		Culture/PCR method (No. of positives)			Primary enrichment (No. of positives)			Secondary enrichment (No. of positives)		
		*S.aureus*	*mecA*	PVL	*S.aureus*	*mecA*	PVL	*S.aureus*	*mecA*	PVL
Animals										
Cow	14	0	0	0	4	0	0	3	0	0
Pig	28	21	0	0	25	0	0	24	1	0
Sheep	35	11	0	0	14	1	0	14	0	0
Total	77	32	0	0	43	1	0	41	1	0
Meat										
Beef	36	9	0	0	10	0	0	12	0	0
Pork	37	25	2	0	26	6	0	27	6	0
Poultry	39	24	0	0	28	0	0	21	0	0
Total	112	58	2	0	64	6	0	60	6	0
Deli meat										
Chicken	9	2	0	0	2	0	0	4	0	0
Ham	20	3	0	0	2	3	0	11	5	0
Turkey	16	0	0	0	0	1	0	4	1	0
Total	45	5	0	0	4	4	0	19	6	0
Total	234	95	2	0	111	11	0	120	13	0

By the conventional culture/PCR method alone, the rate of positive *S. aureus* samples was found to be 41.6% (CI95%: 30.6–52.6%) in animals and 51.8% (CI95%: 42.5–61.0%) in raw meat samples respectively; a significantly lower rate of 11.1% (CI95%: 4.5–24.1%) was observed in deli meat (*P*≤0.05). Using the primary enrichment samples and real-time PCR, a significantly higher recovery of *S. aureus* (*P*≤0.05) was found in animals (55.8%, CI95%: 44.8–66.9%) and raw meat (57.1%, CI95%: 47.9–66.3%) than in deli meat samples (8.9%, CI95%: 3.1–21.4%). However, no significant difference (*P*>0.05) was found between the rate of positive *S. aureus* samples in animals (53.2%, CI95%: 42.1–64.4%), raw meat (53.6%, CI95%: 44.3–62.8%) and deli meat (42.2%, CI95%: 27.8–56.7%), when the secondary enrichment samples were tested by real-time PCR. A significantly higher recovery of *S. aureus* (*P*≤0.05) was obtained from deli meat when the secondary enrichment samples were assessed by real-time PCR.

The *mecA* gene was detected in two pork samples (5.4%, CI95%: 0.7–18.8%) by the conventional multiplex PCR preceded by the culture method, and by assessing the primary and secondary enrichment samples by real-time PCR. The real-time PCR analysis detected the *mecA* gene using both enrichments in samples that were negative by conventional multiplex PCR in two pork meat and three deli meat samples. Using the primary enrichment, the real-time PCR detected the *mecA* gene in one sample isolated from a sheep, and one from pork meat, which were negative using the secondary enrichment. Using the secondary enrichment, the real-time PCR detected the *mecA* gene from one sample isolated from a pig, one from pork meat, and two from deli meat, which were negative using the primary enrichment.

The PVL gene was not detected in any sample by the conventional culture/PCR method or the real-time PCR assay.

Table 3 shows the results of real-time PCR using primary and secondary enrichments on the detection of *S. aureus* compared with a conventional culture/PCR method. Total agreement and the *kappa* statistic for real-time PCR using the primary enrichment samples were 85.7% (*k* = 0.72, CI95%: 0.62–0.82), 83.9% (*k* = 0.68, CI95%: 0.59–0.76), and 97.8% (*k* = 0.88, CI95%: 0.78–0.97) for animals, raw meat, and deli meat respectively. For real-time PCR using the secondary enrichment samples, the total agreement and the *kappa* statistic were 88.3% (*k* = 0.77, CI95%: 0.67–0.86), 87.5% (*k* = 0.75, CI95%: 0.67–0.83), and 68.9% (*k* = 0.29, CI95%: 0.16–0.43) for animals, raw meat, and deli meat respectively. Positive agreement (sensitivity) was 100% for animal samples using both enrichments. For animals and raw meat, a higher negative agreement (specificity) was obtained for real-time PCR using the secondary enrichment.

**Table 3:** Raw agreement indices among conventional culture/PCR method and real-time PCR assay, with two-step enrichment procedure for detection of *S. aureus* from animals and retail meat.

Comparison within each sample type	No. of samples	No. positive by culture/PCR method	No. (%) of samples*			*kappa* statistic
			Positive agreement (Sensitivity)	Negative agreement (Specificity)	Total agreement	
Real-time PCR primary enrichment						
Animals	77	32	32 (100.0)	34 (75.6)	66 (85.7)	0.72
Meat	112	58	52 (89.7)	42 (77.8)	94 (83.9)	0.68
Deli meat	45	5	4 (80.0)	40 (100.0)	44 (97.8)	0.88
Real-time PCR secondary enrichment						
Animals	77	32	32 (100.0)	36 (80.0)	68 (88.3)	0.77
Meat	112	58	52 (89.7)	46 (85.2)	98 (87.5)	0.75
Deli meat	45	5	5 (100.0)	26 (65.0)	31 (68.9)	0.29

*Percentages for positive agreement with culture/PCR method number positive as the denominator. Percentages for negative agreement with culture/PCR method number negative as the denominator. Percentage total agreement is obtained from the sum of the positive and negative agreement frequencies divided by the total sample size within each sample type.

Six samples isolated from animals and six from raw meat were deemed *S. aureus* negative by the conventional culture/PCR method, but positive by real-time PCR using the primary and secondary enrichments. Three *S. aureus* samples isolated from raw meat were positive by the conventional culture/PCR method, but negative by the real-time PCR assay.

The real-time PCR method using the primary enrichment failed to detect the presence of *S. aureus* in four samples: three isolated from raw meat (two from beef, one from poultry) and one from deli meat (ham) that were positive by the culture method and by the real-time PCR assay using the secondary enrichment samples. Using the secondary enrichment samples, the real-time PCR assay failed to detect three samples isolated from raw meat (pork) that were *S. aureus* positive by the culture method and using the primary enrichment in real-time PCR.

The results of real-time PCR using primary and secondary enrichment on the detection of the *mecA* gene compared with a conventional culture/PCR method are shown in Table 4. Total agreement for real-time PCR using the primary and secondary enrichment samples ranged from 91.1% to 98.7% and from 86.7 to 98.7%, respectively. The *kappa* statistic was zero when the *mecA* gene was not detected by the conventional culture/PCR method and 0.49 (CI95%: 0.39–0.58) for raw meat. Positive agreement (sensitivity) of 100% was obtained for raw meat samples for both methods.

**Table 4:** Raw agreement indices among conventional culture/PCR method and real-time PCR assay, with two-step enrichment procedure for detection of the *mecA* gene from animals and retail meat.

Comparison within each sample type	No. of samples	No. positive by culture/PCR method	No. (%) of samples*			*kappa* statistic
			Positive agreement (Sensitivity)	Negative agreement (Specificity)	Total agreement	
Real-time PCR primary enrichment						
Animals	77	0	**-**	76 (98.7)	76 (98.7)	0.00
Meat	112	2	2 (100.0)	106 (96.4)	108 (96.4)	0.49
Deli meat	45	0	**-**	41 (91.1)	41 (91.1)	0.00
Real-time PCR secondary enrichment						
Animals	77	0	**-**	76 (98.7)	76 (98.7)	0.00
Meat	112	2	2 (100.0)	106 (96.4)	108 (96.4)	0.49
Deli meat	45	0	**-**	39 (86.7)	39 (86.7)	0.00

*Percentages for positive agreement with culture/PCR method number positive as the denominator. Percentages for negative agreement with culture/PCR method number negative as the denominator. Percentage total agreement is obtained from the sum of the positive and negative agreement frequencies divided by the total sample size within each sample type.

The real-time PCR assay detected the *mecA* gene in samples that were negative for *S. aureus* by the conventional culture/PCR method (one from a pig, one from a sheep, four from pork, four from deli ham, and one from deli turkey). All of these samples were identified as harboring *S. epidermidis*, *S. saprophyticus* or *S. haemolyticus* using biochemical analysis on isolates recovered. However, three of these samples (one from a pig, two from pork meat) tested positive for the *nuc* gene when primary and secondary enrichments were assessed by real-time PCR.

Table 5 shows the antimicrobial resistance profiles and the sequence types of the ninety-five *S. aureus* strains isolated from animals and retail meat by the conventional culture/PCR method. A total of thirteen antimicrobial resistance profiles were identified among *S. aureus* isolates. Most of the *S. aureus* isolates were resistant to tetracycline and lincomycin, and were of ST9. A total of twenty-two *S. aureus* isolates exhibited multi-drug resistance. Susceptibility to all antimicrobials tested were found in thirty-five *S. aureus* isolates, which were mostly recovered from chicken meat and identified as ST5.

**Table 5:** Antimicrobial resistance profiles and sequence types of *S. aureus* isolated by conventional culture/PCR method from animals and retail meat.

Antimicrobial resistance profile*	No. of antimicrobial subclasses	No. of *S. aureus* isolates with the specific profile	Sequence types (n)†
PEN-TET-ERY-TYL-LINC-STR-CHL	6	2	Pig-ST9 (2)
PEN-TET-LINC-STR-CHL	5	1	Pig-ST9 (1)
TET-ERY-TYL-LINC	3	7	Pork-ST398 (5) Pork-ST5** Pork-ST9 (1)
PEN-LINC-STR	3	1	Pig-ST9 (1)
TET-ERY-LINC	3	7	Pork-ST9 (4) Pork-ST15 (2) Pork-ST8 (1)
TET-LINC-STR	3	1	Pig-ST9 (1)
ERY-TYL-LINC	2	3	Chicken-ST5 (3)
PEN-ERY	2	3	Pork-ST5 (1) Pork-ST5 (1)** Pork-ST9 (1)
TET-LINC	2	15	Sheep-ST398 (4) Pig-ST9 (11)
ERY-LINC	2	1	Pork-ST9 (1)
TET	1	13	Sheep-ST398 (3) Sheep-ST133 (2) Sheep-ST2111 (1) Pig-ST9 (1) Pork-ST1 (2) Pork-ST5 (2) Pork-ST398 (1) Pork-ST15 (1)
ERY	1	1	Deli chicken Chicken-ST39 (1)
LINC	1	5	Pig-ST9 (3) Sheep-ST133 (1) Deli ham-ST15 (1)
Susceptible to all tested	0	35	Chicken-ST5 (15) Chicken-ST6 (3) Chicken-ST508 (1) Chicken-NT‡(1) Pork-ST5 (2) Beef-ST1159 (3) Beef-ST2187 (1) Beef-ST188 (1) Beef-ST15 (1) Beef-ST72 (1) Beef-ST5 (1) Beef-ST1 (1) Deli ham-ST146 (1) Deli ham-ST5 (1) Deli chicken-ST5 (1) Pig-ST9 (1)
Total		95	

*Antimicrobial abbreviations are as following: CHL, chloramphenicol; ERY, erythromycin; LINC, lincomycin; PEN, penicillin; STR, streptomycin, TET, tetracycline, TYL, tylosin.

† ST, sequence type.

‡ NT, non-typeable.

**mecA gene positive.

## Discussion

In this study, a high recovery of *S. aureus* was found in animals and meat samples by the culture/PCR method and the real-time PCR assay (Table 2). The inclusion of selective enrichment steps has been found to increase the rate of detection of *S. aureus* [15]. Waters *et al*. (2011) [26] also found a high prevalence of *S. aureus* in raw meat (47%) using a single step selective enrichment protocol, followed by plating on Baird Parker agar, and confirmation by real-time PCR targeting the *femA* gene.

The *kappa* statistic for detection of *S. aureus* using the primary enrichment in real-time PCR was 0.68–0.88 (Table 3), which indicates a good agreement (substantial to almost perfect agreement) with the conventional culture/PCR method. Using the secondary enrichment and real-time PCR, the *kappa* statistic for detection of *S. aureus* was 0.29–0.77, resulting in a fair agreement when deli meat was tested. This is due to the significantly higher recovery of *S. aureus* from the secondary enrichment samples by real-time PCR (Table 2), and the lower negative agreement (specificity) obtained with this method (Table 3). This observation suggests that small numbers (or levels) of *S. aureus* could be missed when the primary enrichment alone is used in real-time PCR, and that the recovery of potentially injured or non-viable strains appears to be enhanced when a secondary enrichment is applied. The enhanced detection also suggests that the use of a standard culture method or primary enrichment alone could lead to higher false negative results. Therefore, including a secondary selective enrichment step appears to improve the odds of detection of positive *S. aureus* samples.

Multiplex real-time PCR could detect more *S. aureus* positive samples than the conventional culture/PCR method alone. Possible reasons for these discrepant results include: amplification of DNA by the real-time PCR from very low levels of *S. aureus* that were not detectable by the bacteriological methods due to competition or non-viable *S. aureus* in the samples, or false-positive real-time PCR results as a result of cross-reaction rather than false-negative culture results [23]. However, the possibility that these results are considered as false positives in this study is probably very low, because the gene *nuc*, which was targeted by the real-time PCR assay, has been used for specific detection and identification of *S. aureus* previously [21], [22], [34], [35]. Unfortunately, it was not possible to confirm these results by performing the cultural method as detection was carried out from DNA extracts only, and the cells had already been inactivated. The inability of real-time PCR to detect three *S. aureus* samples isolated from raw meat that were positive by the culture method is somewhat unsatisfactory, and could be considered as false-negative results.

For detection of *mecA* gene, the *kappa* statistic for both enrichments in real-time PCR was 0–0.49 (Table 4). The *k* = 0 indicates no agreement beyond that expected by chance, because the real-time PCR assay detected the *mecA* gene probably from bacteria other than *S. aureus* and the culture/PCR method detected the *mecA* gene from DNA extracted from confirmed *S. aureus* strains. However, a few *mecA* positive samples were obtained from animals and meat in this study (Table 2). Weese *et al*. (2010) [25] detected a low prevalence of MRSA in samples isolated from retail meat (9.6% in pork, 5.6% in beef and 1.2% in chicken), using a single-step selective enrichment protocol, followed of plating and biochemical testing.

The detection of the *mecA* gene by the real-time PCR assay in samples that were negative for *S. aureus* by the conventional culture/PCR method may be due to the fact that either coagulase-negative staphylococci and non *S. aureus* species can also carry the *mecA* gene [21], [36]–[38]. In this study, such samples were identified as *Staphylococcus* spp. positive by biochemical testing. In addition, the *mecA* gene has been found in non-staphylococcal genera, such as: *Proteus vulgaris*, *Morganella morganii*, *Enterococcus faecalis* [39] suggesting that its use in a rapid screening technique would need further validation to avoid false-positive MRSA data being generated. In this study, the DNA extraction was carried out from selective enrichments, which could contain DNA from coagulase-positive or coagulase-negative staphylococci or non-staphylococcal species that may carry the *mecA* gene, therefore a positive result for the *nuc* and *mecA* genes does not indicate the presence of *S. aureus* carrying the *mecA* gene.

None of the samples obtained from animals and retail meat were positive for the PVL genes using both methods the conventional multiplex PCR and the real-time PCR. A similar observation was reported by Weese *et al*. (2010) [25], who also failed to detect PVL positive samples in raw meat in Canada using the real-time PCR technique. The PVL genes encode the Panton-Valentine leukocidin toxin, which is a virulence factor that have been found in severe cases of CA-MRSA [7], [8], [9].

Decreasing the time of detection of *S. aureus* and MRSA has become an important goal in microbiological analysis of clinical samples. However, since *S. aureus* ST398, multi-drug resistant *S. aureus* (Table 5), and MRSA are present in animals and meat [12]–[17], decreasing the time of analysis may allow for prompt action to take place thus reducing the spread of those strains in the food chain. The real-time PCR assay can potentially decrease the total time for detection of *S. aureus* and the presence of the *mecA* gene in animal and meat samples. Using the two-step selective enrichment the total time was <2 days by the real-time PCR method, compared with a total time of 6–7 days using the culture method that includes selective enrichments, plating steps, biochemical testing and a conventional multiplex PCR for confirmation. However, the presence of MRSA should be confirmed by a culture method if isolates are required for follow on studies. Some real-time PCR assays have been developed for the rapid detection of MRSA from clinical samples [37], [40]–[42]. Danial *et al*. (2011) [42] reported that the real-time PCR assay detected 0.7% more MRSA-positive samples than the routine standard Brilliance Chromogenic MRSA agar culture method in a total time of 8 h. Huletsky *et al*. (2004) [40] detected MRSA directly from clinical specimens containing a mixture of staphylococci in less than 1 h, with a false-positive detection rate of 4.6% for MRSA that was actually MSSA. Paule *et al*. (2005) [41] developed a multiplex real-time PCR that detected the genes *femA* and *mecA* directly from blood culture bottles in 2–3 h, obtaining an indeterminate rate of 0.9% when coagulase-negative staphylococci strains were included.

In conclusion, the application of real-time PCR using selective enrichments appears to improve the detection of *S. aureus* and the *mecA* gene in samples extracted from animals, raw meat and deli meat. The real-time PCR assay may be recommended as a rapid method to detect *S. aureus* and the *mecA* gene in samples obtained from the meat production chain; however, if further confirmation of MRSA should be required (isolate recovery) then the application of the standard culture method in parallel may be warranted.
